# Supplementary material for: Identification of photoperiod-induced specific miRNAs in the adrenal glands of Sunite sheep (Ovis aries)
Source: Front Vet Sci. 2022 Jul 22;9:888207. doi: 10.3389/fvets.2022.888207 (PMC9354845; doi:10.3389/fvets.2022.888207)
Supplement: Supplementary file 1 [file Data_Sheet_1.ZIP › Supplementary file/miRDeep2 file.zip!/miRDeep2 file/Know_Structure/LP42A1/oar-mir-3956.pdf]

miRBase precursor : oar-mir-3956
Total read count : 75
oar-miR-3956-5p read count : 71
oar-miR-3956-3p read count : 4
remaining reads : 0

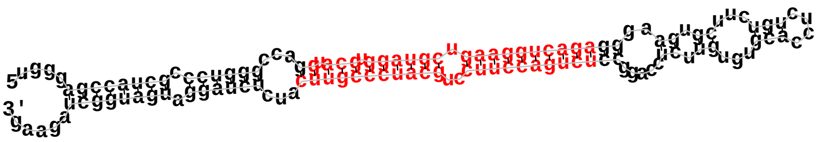

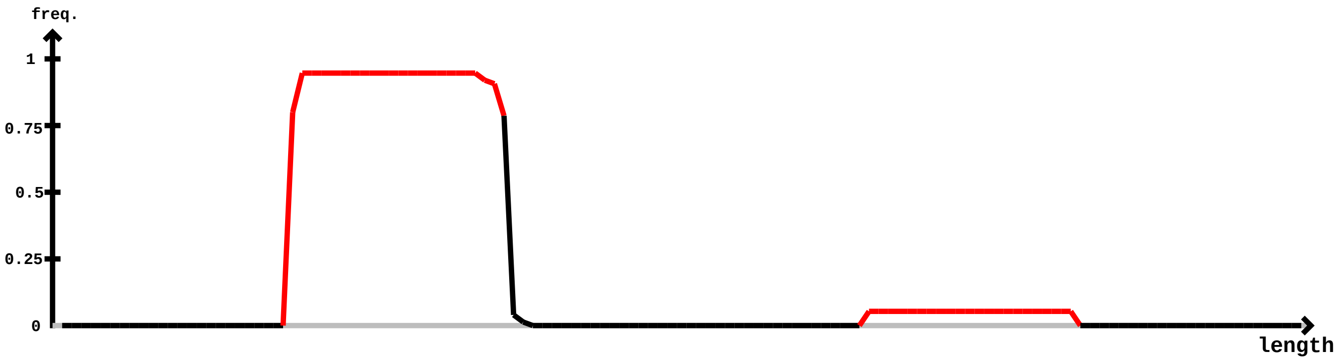


```
                                                                                    oar-miR-3956-3p
                        oar-miR-3956-5p
5'- ugggagccaucgcccugggccagguacguggaugcugaaggucagagggaagugcuucugucuccacguguguucuccaggccucugaccuuccugcaucccguucaucucuaggaugauggcuagaag  -3'   exp
    ....((((((((.((((((...((.(((.((((((.(((((((((((((..((.((...(((....)))...)).)).....)))))))))))))..)))))))))))...)))))).)))))))).....    reads   mm
    .......................guacguggaugcugaagguc.......................................................................................    2       0
    .......................guacguggaugcugaaggucag.....................................................................................    8       0
    .......................guacguggaugcugaaggucaga....................................................................................    49      0
    .......................guacguggaugcugaaggucagagg..................................................................................    1       0
    ........................uacguggaugcugaagguca......................................................................................    1       0
    ........................uacguggaugcugaaggucag.....................................................................................    1       0
    ........................uacguggaugcugaaggucaga....................................................................................    7       0
    ........................uacguggaugcugaaggucagag...................................................................................    2       0
    ...................................................................................ucugaccuuccugcaucccguu........................    4       0
```
